# Genotype -phenotype correlation of *MEFV* mutations in Eastern / Central European population

**DOI:** 10.1186/1546-0096-13-S1-P100

**Published:** 2015-09-28

**Authors:** N Toplak, M Debeljak, T Avcin

**Affiliations:** 1 University Children's Hospital Ljubljana, Department of Allergology, Rheumatology and clinical Immunology, 1000 Ljubljana, Slovenia; 2 Faculty of Medicine, Ljubljana, Ljubljana, Slovenia; 3 Unit for special laboratory diagnostics, University Children's Hospital, University Medical Center, Ljubljana, Slovenia

## Introduction

Familial Mediterranean fever (FMF) is a rare disease in Central and Eastern European countries and phenotype -genotype correlation in this population is not well established.

## Objectives

To study phenotype of patients with *MEFV* mutations and periodic fevers or any other periodic complaints in Eastern / Central European population.

## Methods

Patients who tested positive for *MEFV* mutations were included. Age at disease onset, delay to diagnosis, clinical picture and *MEFV* mutations were collected. Genetic testing was performed in the Genetic laboratory of University Children's Hospital Ljubljana. All 10 exons and exon/intron regions of *MEFV* gene were directly sequenced with ABI Prism 310 Genetic analyzer.

## Results

Fifteen patients (8 male, 7 female) from Eastern / Central European population with median age at disease onset 4 years (range 1.5-17) and median age at *MEFV* testing 8 years (range 3-17) were included; 9/15 patients with mutations in exon 10 (M694V, K695R, A744S, S730F), 3/15 with mutations in exon 3 (P369S/R408E, P369S/R408Q) and 3/15 with mutations in exon 2 (E148Q, A289E). One patient had 2 mutations; in exon 9 (I591T) and exon 10 (K695R).

Variant K695R, found in 6 patients, was associated with a very heterogeneous clinical manifestations; 3 patients presented as PFAPA, 1 patient had clinical features of systemic juvenile idiopathic arthritis and macrophage activation syndrome, 1 patient presented with myelodysplastic syndrome and periodic fevers and later developed acute lymphoblastic lymphoma and one patient who was compound heterozygote for K659R and I591T presented with recurrent pericardial effusions, fever and at first attack at the age of 5 years appendicitis and hemophagocytic lymphohistiocytosis.

Variant M694V was found in 1 patient with typical FMF picture. Variant A744S was found in 1 patient with recurrent pericardial effusions and fever. Variant S730F, a novel variant, was found in one patient with severe recurrent abdominal pains, vomiting and arthralgia without fever.

Variant P369S/R408Q, found in 2 patients, variant A289E, found in 1 patient and variant E148Q, found in 1 patient, were associated with clinical features of PFAPA. One patient with variant E148Q had persistent abdominal pain. Variant P369S/R408E was found in 1 patient with recurrent abdominal pain without fever.

## Conclusion

Patients from Eastern / Central European population with MEFV mutations presented with very heterogeneous clinical phenotypes. To better define clinical presentation of patients with *MEFV* mutations genetic testing should be performed also in cases with inconclusive FMF clinical presentation.

